# Two RNase H2 Mutants with Differential rNMP Processing Activity Reveal a Threshold of Ribonucleotide Tolerance for Embryonic Development

**DOI:** 10.1016/j.celrep.2018.10.019

**Published:** 2018-10-30

**Authors:** Ryo Uehara, Susana M. Cerritelli, Naushaba Hasin, Kiran Sakhuja, Mariya London, Jaime Iranzo, Hyongi Chon, Alexander Grinberg, Robert J. Crouch

**Affiliations:** 1 SFR, Division of Intramural Research, Eunice Kennedy Shriver National Institute of Child Health and Human Development, NIH, Bethesda, MD, USA; 2 NCBI, National Library of Medicine, Bethesda, MD, USA; 3 Mouse Core, Division of Intramural Research, Eunice Kennedy Shriver National Institute of Child Health and Human Development, NIH, Bethesda, MD, USA; 4 Lead Contact

## Abstract

RNase H2 has two distinct functions: initiation of the ribonucleotide excision repair (RER) pathway by cleaving ribonucleotides (rNMPs) incorporated during DNA replication and processing the RNA portion of an R-loop formed during transcription. An RNase H2 mutant lacking RER activity but supporting R-loop removal revealed that rNMPs in DNA initiate p53-dependent DNA damage response and early embryonic arrest in mouse. However, an RNase H2 AGS-related mutant with residual RER activity develops to birth. Estimations of the number of rNMPs in DNA in these two mutants define a ribonucleotide threshold above which p53 induces apoptosis. Below the threshold, rNMPs in DNA trigger an innate immune response. Compound heterozygous cells, containing both defective enzymes, retain rNMPs above the threshold, indicative of competition for RER substrates between active and inactive enzymes, suggesting that patients with compound heterozygous mutations in *RNASEH2* genes may not reflect the properties of recombinantly expressed proteins.

## In Brief

Uehara et al. use RNase H2 mice with differing activity levels for removal of rNMPs embedded in DNA. Moderate levels of rNMPs lead to perinatal lethality activating the cGAS-Sting DNA sensing innate immune response. Exceeding a threshold, high abundance of rNMPs activates p53-dependent DNA damage, causing early embryonic lethality.

## Graphical Abstract

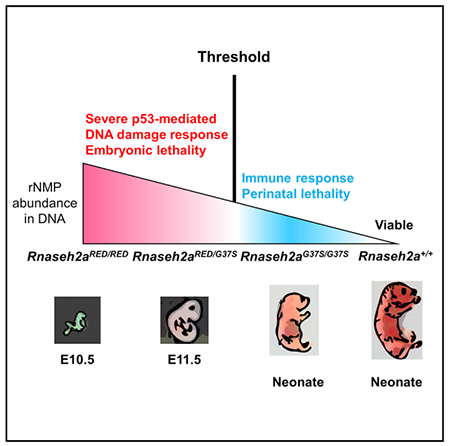

## INTRODUCTION

Errors during DNA replication and repair processes can challenge genome integrity. Cells are well equipped to restore normal duplex DNA, but in some circumstances defects may persist, leading to severe DNA damage. Discrimination against rNTPs by DNA polymerases at the sugar moiety is far from perfect, and the three replicative DNA polymerases α, δ, and ε include ribonucleotide monophosphates (rNMPs) at different frequencies, resulting in about 1 rNMP for every 7,000 deoxyribo-nucleotide monophosphates (dNMPs) in DNA in mouse (Hiller et al., 2012; Reijns et al., 2012) and yeast (Lujan et al., 2012; Williams et al., 2016) cells. The abundance of rNMPs incorporated in DNA is significantly higher than any of the well-studied modified nucleotides produced by, for example, UV irradiation. The highly efficient ribonucleotide excision repair (RER) pathway is initiated by incision at the rNMP by RNase H2 followed by repair synthesis to replace rNMPs by dNMPs (Sparks et al., 2012). From a biochemical standpoint, rNMP incorporation could be described as a replication error, but because these rNMPs are included so frequently and efficiently removed, we and others have suggested that biologically, the transient presence of rNMPs in DNA serves a positive role in DNA replication (Cerritelli and Crouch, 2016; Clausen et al., 2015), perhaps as a mark for mismatch repair as suggested (Ghodgaonkar et al., 2013; Lujan et al., 2012) or to release torsional constrains. RNase H2 is uniquely suited to recognize rNMPs in DNA, but it also cleaves the RNA strand of RNA/DNA hybrids mostly found in cells as part of R-loops (with the non-hybridized DNA in single-stranded form) (Cerritelli and Crouch, 2009). R-loops, which form during transcription, are also processed by RNase H1 (Drolet et al., 1995). Complete absence of RNase H2 eliminates both activities, making it difficult to assign any phenotype to defects in RER or RNA/DNA hydrolysis. We modified the Rnh201 catalytic subunit in yeast RNase H2 to retain RNA/DNA activity, eliminating almost completely its rNMP cleavage activity, resulting in an RNase H2^RED^ (ribonucleotide excision defective) (Chon et al., 2013). We verified that typical 2–5 bp deletions created in the absence of RNase H2 were as prevalent in the RNase H2^RED^ mutant. The RNA/DNA hybrid activity of RNase H2^RED^ rescued the growth defect of *top1 Δrnh1 Δrnh201 Δ* strain and the synthetic slow growth of *sgs1 Δrnh201 Δ* strain, both indicative of defects in RNA/DNA processing.

RNase H2 is essential in mice and medically relevant in humans. More than half of patients with the neuroinflammatory Aicardi-Goutières syndrome (AGS) have biallelic mutations in any of the three RNase H2 subunits (Crow et al., 2015). Some AGS-related *RNASEH2* mutations have been associated with systemic lupus erythematosus (Günther et al., 2015; Ramantani et al., 2010). Both RER and RNA/DNA hydrolysis activities are absent in mouse in which RNase H2-B or H2-C subunit is ablated, resulting in embryonic lethality at around E10.5 (Hiller et al., 2012; Reijns et al., 2012). It seems reasonable that RNase H2’s unique ability to cleave at single rNMPs in DNA is directly related to developmental arrest in the absence of RNase H2. Reijns et al. (2012) drew such a conclusion, and others (Günther et al., 2015) suggested that the DNA damage caused by unrepaired rNMPs is the main contributor to AGS. However, Chedin’s laboratory examined cell lines from several AGS patients and attributed AGS phenotypes to unresolved R-loops (RNA/DNA hybrids) (Lim et al., 2015).

To examine the contribution of each of RNase H2 activities in mice to viability and genome integrity, we used three *Rnaseh2a* mutant mice: knockout; *Rnaseh2a^G37S^*, a hypomorphic mutation (Pokatayev et al., 2016) producing an enzyme with partial loss of both RNA/DNA hydrolysis and RER; and *Rnaseh2a^RED^*, which is ribonucleotide excision defective but active for RNA/DNA hybrid processing. By crossing these three mice with different RNase H2 activities, we observed that accumulation of rNMPs triggered activation of the p53-mediated DNA damage response pathway in *Rnaseh2a^RED/RED^*, *Rnaseh2a*^*RED*/−^ and *Rnaseh2a*^−/−^ embryos, resulting in early embryonic developmental arrest. This strongly indicates that the abundance of unrepaired rNMPs is the cause of embryonic lethality. We also found that although *Rnaseh2a*^*G37S*/−^ mice survived to birth, they became embryonic lethal at E12.5 in *Rnaseh2a^G37S/RED^* mice, suggesting that the RNase H2^RED^ enzyme acts in a dominant manner for limiting the RER pathway. Such differences in activity among RNase H2 mutants may be relevant for AGS patients with biallelic mutations (Crow et al., 2015), which could present complex defects due to competitive binding on genomic DNA substrates.

## RESULTS

We expressed different variants of RNase H2 in mice to learn how its two activities contribute to viability and fitness. A mutant strain in which RNase H2^RED^ is the sole source of the enzyme is the centerpiece of the results we report here.

### *In Vitro*, Mouse RNase H2^RED^ Is Unable to Remove Single Ribonucleotides in dsDNA but Retains High Levels of RNA/DNA Degradation Activity

**Figure 1.**
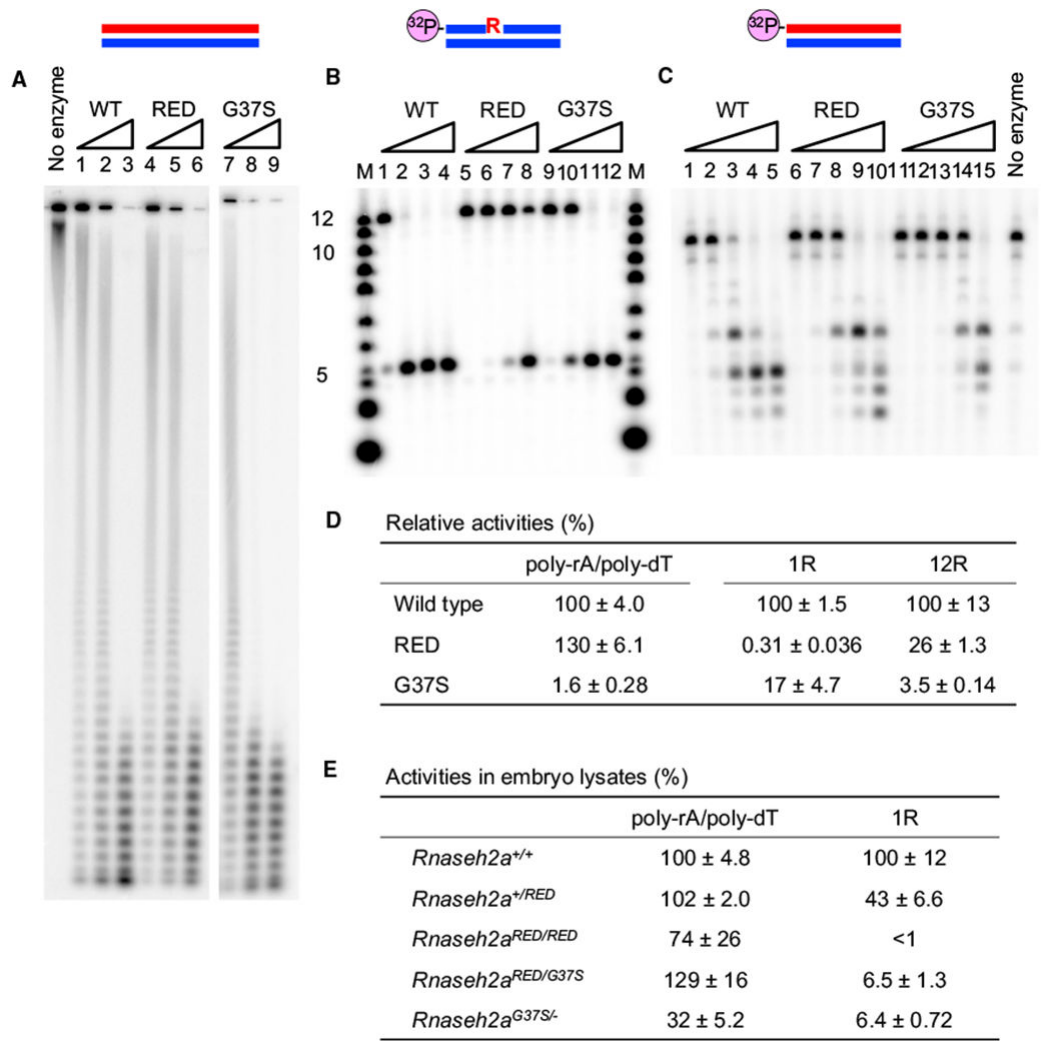
*In Vitro* Activities of RNase H2^WT^, RNase H2^RED^, and RNase H2^G37S^ (A) The uniformly ^32^P-labeled poly-rA/poly-dT substrate (1 μM) was hydrolyzed with increasing amounts of RNase H2^WT^ and its derivatives at 37°C for 30 min. Lanes 1 and 4 contained 10 pM proteins, lanes 2 and 5 contained 20 pM proteins, lanes 3 and 6 contained 50 pM proteins, lane 7 contained 2 nM protein, lane 8 contained 5 nM protein, and lane 9 contained 10 nM protein. Reaction products were analyzed by 12% TBE-urea PAGE. (B) The 5′-^32^P-labeled 12-mer 1R (DNA_5_-RNA_1_-DNA_6_/DNA_12_) substrate (1 μM) was hydrolyzed with increasing amounts of RNase H2 and its derivatives at 37°C for 30 min. Lanes 1,5, and 9 contained 100 pM proteins; lanes 2,6, and 10 contained 1 nM proteins; lanes 3, 7, and 11 contained 10 nM proteins; and lanes 4,8, and 12 contained 100 nM proteins. Reaction products were analyzed by 20% TBE-urea PAGE. The molecular size marker is indicated as M. (C) The 5′-^32^P-labeled 12-mer 12R (RNA_12_/DNA_12_) substrate (1 μM) was hydrolyzed with increasing amounts of RNase H2 and its derivatives at 37°C for 30 min. Lanes 1,6, and 11 contained 10 pM proteins; lanes 2, 7, and 12 contained 100 pM proteins; lanes 3, 8, and 13 contained 1 nM proteins; lanes 4, 9, and 14 contained 10 nM proteins; and lanes 5, 10, and 15 contained 100 nM proteins. Reaction products were analyzed by 20% TBE-urea PAGE. (D) Specific activities of mutant enzymes relative to wild-type activity (%). (E) Relative RNase H2 activities (%) in E11.5 embryos lysates. The mean values of three independent embryos are shown with SD. Enzymatic activities were normalized to the mean value of uterine sibling wild-type embryos.

We wanted to confirm that mouse RNase H2A protein with the corresponding substitutions of the RED mutant (P40D and Y211A) exhibits the same enzymatic characteristics as the *S. cerevisiae* RNase H2^RED^ enzyme. We purified the mutated enzyme expressed in *E. coli* to near homogeneity, detecting only the three expected protein bands of the RNase H2 subunits by SDS-PAGE (Figure S1A). We examined enzymatic activities of mouse RNase H2^RED^ using uniformly ^32^P-labeled poly-rA/poly-dT (hereafter noted as Hybrid) (Figure 1A) and 5′ end-labeled DNA 12 bases in length containing a single rNMP 1R (DNA_5_-RNA_1_DNA_6_/DNA_12_) (Figure 1B) and 12R (RNA_12_/DNA_12_) substrates (Figure 1C). RNase H2^RED^ exhibited 130% ± 6.1% of the activity of the RNase H2^WT^ on hybrid substrates (Figure 1D). The degradation of Hybrid by RNase H2^RED^ was slightly faster than that of RNase H2^WT^. RNase H2^WT^ produced shorter RNA fragments and degraded 12R substrate faster than RNase H2^RED^ (Figure 1C), suggesting that the RNase H2^RED^ enzyme prefers long RNA/DNA hybrids to short substrates, as previously found for the yeast enzyme (Chon et al., 2013). In contrast, RNase H2^RED^ retained only 0.31% ± 0.036% of the RNase H2^WT^ RER activity toward the 1R substrate, demonstrating that mouse RNase H2^RED^ is, like its yeast homolog, an RNA/DNA-specific enzyme. *Rnaseh2a^G37S^* is an AGS-related mutation that impairs the catalytic function of RNase H2 in human and yeast (Chon et al., 2009, 2013; Coffin et al., 2011; Crow et al., 2006; Perrino et al., 2009). We found that mouse RNase H2 with the corresponding mutation (RNase H2^G37S^) retains activities toward Hybrid, 1R, and 12R substrates but greatly reduced to 1.6% ± 0.28%, 17% ± 4.7%, and 3.5% ± 0.14% of wild-type, respectively (Figure 1D).

### *Rnaseh2a* KO Mice Are Early Embryonic Lethal

**Figure 2.**
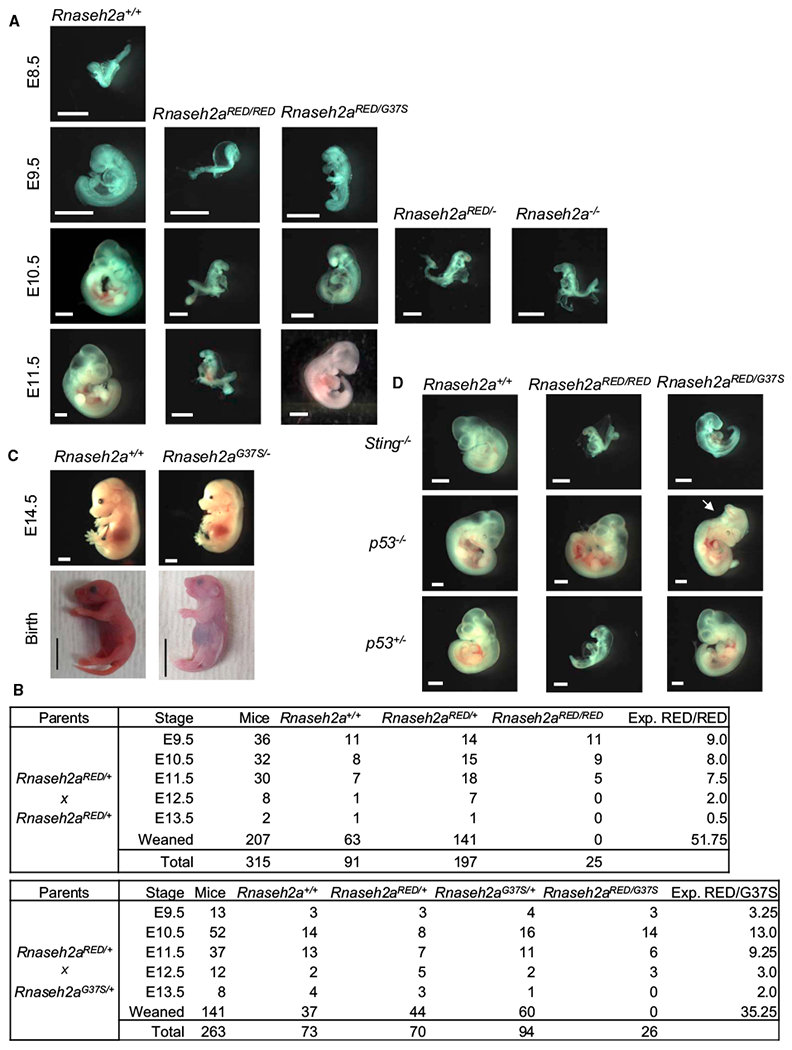
Developmental Defect of Embryos (A) *Rnaseh2a*^+/+^, *Rnaseh2a^RED/RED^*, *Rnaseh2a^RED/G37S^*, *Rnaseh2a*^*RED*/−^, and *Rnaseh2a*^−/−^ embryos at embryonic days 8.5–11.5. Scale bar, 1 mm. (B) Survival of mouse embryos from crosses of *Rnaseh2a*^*RED*/+^ × *Rnaseh2a*^*RED*/+^ (top) and *Rnaseh2a*^*RED*/+^ × *Rnaseh2a*^*G37S*/+^ (bottom). Expected number of *Rnaseh2a^RED/RED^* and *Rnaseh2a^RED/G37S^* at Mendelian ratio is shown in right column. (C) *Rnaseh2a*^+/+^ and *Rnaseh2a*^*G37S*/−^ embryos and neonates. Scale bars, 2 mm (E14.5) and 1 cm (birth). (D) Partial rescue of embryonic lethality by ablation of *p53* gene. Images of E10.5 embryos are shown. A morphological rescue of *Rnaseh2a^RED/RED^* and *Rnaseh2a^RED/G37S^* was observed on *p53* background. *Rnaseh2a^RED/G37S^* was also rescued on *p53*^+/−^ background (n = 5), whereas *Rnaseh2a^RED/RED^* was not (n = 3). No rescue of *Rnaseh2a^RED/RED^* (n = 8) and *Rnaseh2a^RED/G37S^* (n = 5) phenotypes was observed on *Sting*^−/−^ background.

We wished to examine mice homozygous and heterozygous for RNase H2-defective mutations in the *Rnaseh2a* gene. We obtained *Rnaseh2a^tm1(KOMP)Wtsi^* knockout (KO) embryonic stem cells (ESCs) from the Mouse Knockout Project and by standard techniques generated viable heterozygous mice. *Rnaseh2a*^−/−^ embryos arrested in development at about E10.5 (Figure 2A), consistent with results observed for inactivation of the *Rnaseh2b* or *Rnaseh2c* genes (Hiller et al., 2012; Reijns et al., 2012).

### Embryonic Lethality of *Rnaseh2a^RED/RED^*

Inactivation of any of the three genes encoding RNase H2 subunits leads to complete loss of both RER and Hybrid activities. We wanted to address directly the role of RER activity in embryonic viability in mice by using a knock-in mouse strain carrying an *Rnaseh2a* allele that exclusively abolishes RER activity, as we demonstrated *in vitro* for the recombinant enzyme (see above and Figure 1). We generated a *Rnaseh2a^P40D-Y211A^* (*Rnaseh2a^RED^*) mouse. We obtained viable heterozygous *Rnaseh2a*^*RED*/+^ mice that exhibited no detectable abnormalities. Intercrossing of *Rnaseh2a*^*RED*/+^ mice resulted in live *Rnaseh2a*^*RED*/+^ and wild-type (*Rnaseh2a*^+/+^) mice at a ratio of approximately 2:1, but no viable *Rnaseh2a^RED/RED^* mouse was found in more than 200 weaned mice (Figure 2B). We subsequently investigated embryos from crosses of *Rnaseh2a*^*RED*/+^ parents and observed *Rnaseh2a^RED/RED^* embryos at Mendelian ratios at early stages of development (Figure 2B). At embryonic day 9.5 (E9.5), *Rnaseh2a^RED/RED^* embryos were developmentally retarded by approximately 1 day compared with their littermates (Figure 2A). E10.5 *Rnaseh2a^RED/RED^* embryos were not resorbed yet but remained morphologically abnormal, with a slight increase in size compared with E9.5 embryos. E11.5 embryos appeared unchanged from day E10.5. No E12.5 *Rnaseh2a^RED/RED^* embryo was found. Therefore, *Rnaseh2a^RED/RED^* embryos exhibited delayed development and completely arrested at E10.5 and were resorbed after E11.5. We subsequently crossed *Rnaseh2a*^−/+^ and *Rnaseh2a*^*RED*/+^ mice and compared *Rnaseh2a*^−/−^, *Rnaseh2a*^*RED*/−^, and *Rnaseh2a^RED/RED^* embryos at E10.5. They were indistinguishable in size and tissue morphology (Figure 2A). We conclude that *Rnaseh2a^RED/RED^* and *Rnaseh2a*^*RED*/−^ exhibit the same early embryonic lethality as seen when RNase H2 is completely absent (Hiller et al., 2012; Reijns et al., 2012). In addition, we conclude that a single wild-type *Rnaseh2a* allele is sufficient to produce normal mice and that the presence in the same cells of RNase H2^RED^ mutant enzyme does not influence normal viability, as previously reported for *Rnaseh2a*^+/*G37S*^ mice.

### *Rnaseh2a^G37S^* Contribution to Mouse Development

We previously reported that the hypomorphic RNase H2^G37S^ enzyme allowed *Rnaseh2a^G37S/G37S^* mice to survive only to birth, with a smaller size than the *Rnaseh2a*^*G37S*/+^ and wild-type (WT) mice in the same birthing. *Rnaseh2a^G37S/G37S^* embryos remains proportionally smaller through development (Figure S2A). *Rnaseh2a*^*G37S*/+^ mice are not defective in any obvious way. RNase H2^G37S^ retains RER and Hybrid activities but both are significantly decreased (Figure 1D). When we crossed *Rnaseh2a*^−/+^ and *Rnaseh2a*^*G37S*/+^ mice, we discovered that *Rnaseh2a*^*G37S*/−^ also developed to birth and were similarly retarded in growth only slightly smaller than *Rnaseh2a^G37S/G37S^* dead pups (Figures 2C and S2A). At delivery, *Rnaseh2a^G37S/37S^* mice weighed about 0.9 g compared with 1.2 g for *Rnaseh2a^G37S/G37S^* (each an average of four pups). *Rnaseh2a^G37S/G37S^* and even *Rnaseh2a*^*G37S*/−^ embryonic development goes beyond that of *Rnaseh2a*^*RED*/−^, *Rnaseh2a^RED/RED^*, and *Rnaseh2a*^−/−^ embryos because of the presence of sufficient RNases H2 activities to reduce DNA damage.

### *Rnaseh2a^G37S/RED^* Mice Are Early Embryonic Lethal

We anticipated that a mouse having both RNase H2^G37S^ and RNase H2^RED^ might be viable because of increased Hybrid activity of RNase H2^RED^ and RER activity of RNase H2^G37S^. Surprisingly, we found no viable or perinatal-lethal *Rnaseh2a^G37S/RED^* pups when we crossed *Rnaseh2a*^*G37S*/+^ with *Rnaseh2a*^*RED/+*^ mice; rather, they were early embryonic lethal. However, the *Rnaseh2a^RED/G37S^* embryos survived a bit longer than embryos from *Rnaseh2a*^−/−^ or *Rnaseh2a^RED/RED^* mice (Figures 2A and 2B). At E9.5 the *Rnaseh2a^RED/G37S^* embryos were larger and more developed than *Rnaseh2a^RED/RED^* embryos. We saw progression of three *Rnaseh2a^RED/G37S^* embryos to E12.5 with various sizes at E11.5 and E12.5 stages. However, they arrested development at E11.5 some 7 or 8 days before *Rnaseh2a^G37S/G37S^* and *Rnaseh2a*^*G37S*/−^ become perinatal lethal. We propose that either RNase H2^RED^ is expressed at a higher rate and competes with RNase H2^G37S^ for assembly with the RNase H2-B and H2-C subunits, or that RNase H2^RED^ competes with RNase H2^G37S^ in the RER pathway. We address these possibilities in the following sections.

### RNase H Activities in RNase H2^RED^ and RNase H2^G37S^ Embryo Extracts

Instability of RNase H2^RED^*in vivo* could account for the defects we saw during embryonic development of *Rnaseh2a^RED/RED^* mice. We measured RNase H2 Hybrid and RER activities in crude lysates of E11.5 embryos using Hybrid and 1R substrates. We found that Hybrid activity in *Rnaseh2a^RED/RED^* embryo extracts was high, and RER activity was undetectable (Figure 1E). The relative *ex vivo* activities were quite similar to those we found with recombinantly expressed purified enzymes (Figure 1D), indicating stability and efficient assembly of the mutant enzyme *in vivo*. Additionally, immunoblotting of RNase H2-A and H2-C from mouse embryonic fibroblast (MEF) cell homogenates implied that the cellular amount of RNase H2A and H2C, which forms a 1:1:1 heterotrimer with H2B, is almost equivalent in *Rnaseh2a*^+/+^, *Rnaseh2a*^*RED*/+^, and *Rnaseh2a^RED/RED^* (Figure S2B). These results led us to conclude that loss of RER activity accounts for early arrest of development of *Rnaseh2a^RED/RED^* embryos.

*Rnaseh2a*^*G37S*/−^ mice survive to birth with about 6% of RER activity and 30% of Hybrid activity (Figure 1E). The significantly higher Hybrid activity in *Rnaseh2a*^*G37S*/−^ embryo extracts with respect to recombinantly expressed proteins could be due to stimulation of the RNase H2^G37S^ activity or to RNase H1 degradation of the substrate. The former seems more likely because RNase H1 activity is at best only 15% of that of RNase H2 (Büsen and Hausen, 1975). However, the patterns of products are those produced by both RNases H1 and H2 (Figure S2C). Therefore, we cannot rule out that RNase H1 is contributing to activity in *Rnaseh2a*^*G37S*/−^ embryo extracts.

### RNase H2^RED^ Competes with RNase H2^G37S^ in the RER Pathway

Similar RER activity was found in *Rnaseh2a^RED/G37S^* embryo extracts as in extracts of *Rnaseh2a*^*G37S*/−^ embryos (Figure 1E), although the former arrest development much earlier. Of course, these *ex vivo* assays were performed with excess added substrates. We suggest that *in vivo* RNase H2^RED^ retains Hybrid activity and capability to bind to RER substrates, resulting in blocking the removal of genomic rNMPs. Because *Rnaseh2a^RED/G37S^* embryos survived only modestly further than *Rnaseh2a^RED/RED^* embryos, we predict that RNase H2^RED^ might bind better than RNa-seH2^G37S^ to substrates with a single rNMP embedded in DNA. We first tested for differences in association of the two enzymes to an RER substrate immobilized on the sensor chips using surface plasmon resonance. We found that the WT enzyme bound very well to the substrate, but the RNase H2^RED^ and RNase H2^G37S^ both bound very poorly; so poorly that we were unable to fit the binding curves to those of a 1:1 binding model (Figure S3A). Therefore, we performed competition for incision of a single rNMP in double-stranded DNA (dsDNA) *in vitro* using RNase H2^G37S^ and 10- or 100-fold excess of RNase H2^RED^ or RNase H2^D34A^, the latter being totally inactive because of the substitution of the catalytic aspartate residue by alanine. When added in excess, we found that RNase H2^RED^ or RNase H2^D34A^ neither inhibited nor stimulated RNase H2^G37S^ incision activity in these assays (Figure S3B). Even when we added double mutant RNase H2^D34A-RED^ or RNase H2^D34A-G37S^, we did not observe any inhibition of RNase H2^G37S^ activity. Therefore, we conclude that the enzyme-substrate complexes formed by mutant proteins are unstable and cannot compete for binding and catalysis by RNase H2^G37S^. On the basis of these results, we propose that competition occurs later in the RER pathway involving RNase H2’s interaction with PCNA via its PCNA-interacting peptide located on the RNase H2B subunit (see Discussion and Figure S3C).

### Partial Rescue of Embryonic Lethality of *Rnaseh2a^RED/RED^* and *Rnaseh2a^RED/G37S^* Embryos by *p53*^−/−^ but Not by *Sting*^−/−^

We previously reported that *Rnaseh2a^G37S/G37S^* *p53*^−/−^ mice failed to alter the perinatal lethality of *Rnaseh2a^G37S/G37S^* mice, but we did find that *Rnaseh2a^G37S/G37S^* in a *Sting*^−/−^ background eliminates the innate immune response (Pokatayev et al., 2016). We considered the possibility that RNase H2^G37S^ and perhaps RNase H2^RED^ might lead to induction of the innate immune response in *Rnaseh2a^RED/G37S^* embryos. We obtained embryos of *Rnaseh2a^RED/RED^* and *Rnaseh2a^RED/G37S^* in a *Sting*^−/−^ background and found that these embryos had identical characteristics as embryos in a *Sting*^+/+^ strain (Figure 2D). They all arrest at the same stage and embryos exhibited poor morphology.

In contrast to lack of effect of *Sting*^−/−^, *Rnaseh2a^RED/RED^*, and *Rnaseh2a^RED/G37S^* E10.5 embryos in a *p53*^−/−^ background were normal in size, with some morphological abnormalities (Figure 2C, arrow). *Rnaseh2a^RED/RED^ p53*^+/−^ E10.5 embryos were small and defective (bigger than or same as *p53*^+/+^) but *Rnaseh2a^RED/G37S^ p53*^+/−^ E10.5 embryos were normal size. For *Rnaseh2a^RED/RED^* we saw the same effect of *p53*^−/−^ as reported for *Rnaseh2b-* and *Rnaseh2c*-null embryos (Hiller et al., 2012; Reijns et al., 2012). We conclude that the *p53*^−/−^ background effect is consistent with p53-induced DNA damage response, causing very early lethality.

### p53-Mediated DNA Damage Response Caused by Loss of RER Activity

**Figure 3.**
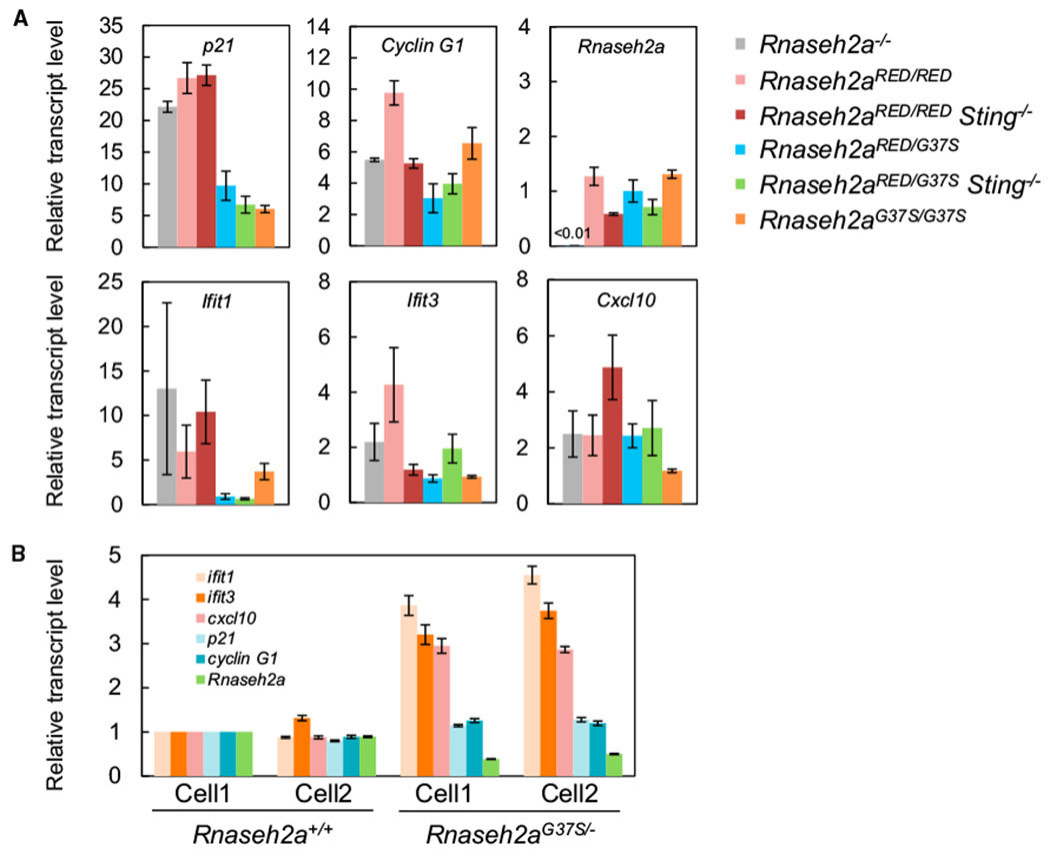
qRT-PCR Analysis of p53-Target and Interferon-Stimulated Genes (A) Expression levels of two p53 target genes (*Cdkn/p21* and *Ccng1/Cyclin G1*) and three interferon-stimulated genes (*Ifit1, Ifit3*, and *Cxcl10*) were measured by RT-qPCR of RNA from E10.5 whole embryos. The mean values of four *Rnaseh2a^RED/RED^*, three *Rnaseh2a*^−/−^, three *Rnaseh2a^G37S/G37S^*, and three *Rnaseh2a^RED/G37S^* embryos were normalized to that of *Rnaseh2a*^+/+^. The mean values of *Rnaseh2a^RED/RED^ Sting*^−/−^ (n = 4) and *Rnaseh2a^RED/G37S^ Sting*^−/−^ (n = 5) embryos were normalized to that of *Rnaseh2a*^+/+^ *Sting*^−/−^. Error bars represent SEM. (B) Expression levels ofp21, cyclin G1, ISGs, and *Rnaseh2a* in *Rnaseh2a*^*G37S*/−^ E14.5 MEFs. MEF cell lines were established from two embryos. Transcript levels were normalized to that of *Rnaseh2a*^+/+^.

Our results, which showed improved growth in embryos from *Rnaseh2a^RED/RED^* and *Rnaseh2a^RED/G37S^* in a *p53*^−/−^ background, demonstrate that p53 response is activated in E10.5 embryos. We determined the expression levels of p53 target genes (*p21* and *Cyclin G1*) by qRT-PCR analyses, and as seen in Figure 3A, *p21* was increased more than 20-fold in *Rnaseh2a*^−/−^, *Rnaseh2a^RED/RED^*, and *Sting*^−/−^ *Rnaseh2a^RED/RED^* embryos but only 6-to 9-fold in *Rnaseh2a^RED/G37S^*, *Sting*^−/−^ *Rnaseh2a^RED/G37S^*, and *Rnaseh2a^G37S/G37S^* embryos. The E10.5 embryos, in which we saw most upregulation, were arresting growth, while the embryos with less *p21* mRNA activation were still in a growth phase. Expression of *cyclin G1* transcripts were also elevated 3- to 10-fold in all embryos, but there was no clear pattern of increases as seen for p21 activation. Furthermore, we found no indication of a cGAS-Sting-dependent immune response for interferon-stimulated genes (ISGs), *Ifit1*, *Ifit3,* and *Cxcl10.* We observed some variations in the expression levels of ISGs, but they were not consistent with the genotypes (e.g., *Ifit1* and *Cxcl10* expression levels in *Rnaseh2a^RED/RED^ Sting*^−/−^ were considerably elevated, whereas *Rnaseh2a^RED/RED^* exhibited moderate upregulation of all three ISGs). We examined the stimulation of these same three ISGs in MEFs from embryos from *Rnaseh2a*^*G37S*/−^ at E14.5 and we saw no activation of *p21* and *CyclinG1* but consistent small increases of the ISG mRNA (Figure 3B). Taking these results together, we concluded that *Rnaseh2a^RED/RED^* and *Rnaseh2a^RED/G37S^* embryos, whether in a *Sting*^−/−^ or *Sting*^+/+^ background, are embryonic lethal because of p53-dependent DNA damage response as reported here also for *Rnaseh2a*^−/−^ and previously for *Rnaseh2b*-null and *Rnaseh2c*^−/−^ embryos (Hiller et al., 2012; Reijns et al., 2012). However, p21 expression in *Rnaseh2a^RED/G37S^* embryos was significantly lower than that of *Rnaseh2a^RED/RED^* embryos, indicative of less DNA damage in the former.

### Increased rNMPs in *Rnaseh2a^RED/RED^*, *Rnaseh2a^RED/G37S^*, and *Rnaseh2a*^*G37S*/−^ MEFs

**Figure 4.**
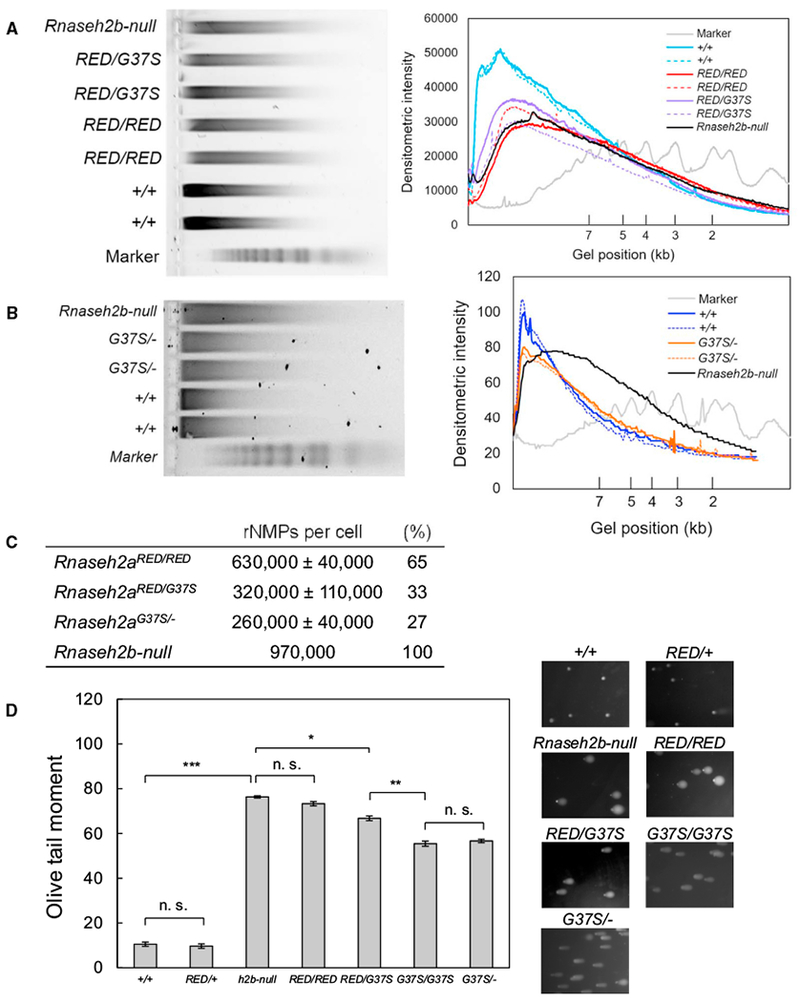
Accumulation of Unrepaired rNMPs Causes Serious DNA Damage (A and B) Differences in abundance of rNMPs in embryonic fibroblast DNA were analyzed using alkaline gel electrophoresis. The genotypes of cell lines are shown in the inset of figures. (A) Increased migration of *Rnaseh2a^RED/RED^* and *Rnaseh2a^RED/G37S^* DNA in alkaline agarose gel. Gel image after electrophoresis and densitometry curves ofeach DNA are shown. Total MEF nucleic acids were isolated, treated with 0.3 M NaOH and analyzed by 0.7% agarose gel electrophoresis in alkaline running buffer. (B) Low but significant number of rNMPs is retained in *Rnaseh2a*^G37S/−^. (C) The estimated numbers of rNMPs in RER-defective cells. (D) Alkaline comet assay of MEFs. Representative images of comets after single-cell electrophoresis are shown on the right. DNA was unwound under alkaline condition followed by electrophoresis. Damaged DNA fragments migrated out of the nucleus, forming a comet tail. DNA damage levels of cells were quantified by olive tail moment and shown in graph. More than 100 comets were randomly chosen and analyzed. The values are expressed in mean ± SEM from three independent experiments. *p < 0.02, **p < 0.005, and ***p < 0.0001 (t test).

To study ribonucleotide presence in DNA of various mutants, we prepared MEFs from embryos at different stages. We readily obtained *Rnaseh2a*^*G37S*/−^ MEFs from E14.5 embryos. In contrast, we were unable to establish MEFs from *Rnaseh2a^RED/RED^* embryos, unless crossed with *p53*^−/−^ mice. We established *Rnaseh2a^RED/G37S^* *p53*^−/−^ cell lines as well. *Rnaseh2a^RED/G37S^* cells in *p53*^+/−^ and *p53*^+/+^ background, like *Rnaseh2a*^RED/RED^, grew poorly in culture medium. To examine the extent to which incorporated ribonucleotides remain in *Rnaseh2a^RED/RED^* *p53*^−/−^ MEFs, we isolated genomic DNA, treated with 0.3 M NaOH, and analyzed by agarose gel electrophoresis at alkaline pH. DNA mobility of *Rnaseh2a^RED/RED^* was increased after alkaline treatment, giving a substantial peak shift of DNA intensity tracing curve from those of WT and *Rnaseh2a*^*RED*/+^ (Figures 4A and S2D). The mobility shift of DNA fragments in alkaline agarose gels for *Rnaseh2b*-null and *Rnaseh2a^RED/RED^* DNA (both *p53*^−/−^ background) with respect to *Rnaseh2a*^+/+^ indicates that a considerable number of rNMPs accumulate in *Rnaseh2b*-null and *Rnaseh2a^RED/RED^* genomic DNA. The number of rNMPs per cell in *Rnaseh2b*-null DNAs was estimated to be about 970,000/cell (Figure 4C), slightly less than reported by Reijns et al. (2012). *Rnaseh2a^RED/RED^* DNA contained about 630,000 ± 40,000 rNMPs/cell, fewer than *Rnaseh2b*^−/−^ but still well over the background strand breaks seen in *Rnaseh2a*^+/+^ MEFs (Figure 4). We suggest that the reason for fewer DNA breaks in *Rnaseh2a^RED/RED^* compared with *Rnaseh2b*^−/−^ cells is the presence of persistent RNA/DNA hybrids in the latter cells, which contribute to replicative stress and DNA damage. However, an alternative explanation could be that the *Rnaseh2a^RED/RED^* cells are early-passage MEFs, whereas the *Rnaseh2b*^−/−^ cells are greater than 20 cell passages and could have accumulated greater numbers of rNMPs. We found large variation of rNMPs frequency in *Rnaseh2a^RED/G37S^* DNA from two independent cell lines, both of which contained significantly fewer rNMPs than *Rnaseh2a*^RED/RED^. In contrast, accumulation of rNMPs in *Rnaseh2a*^*RED*/+^ was undetectable as reported for *Rnaseh2b*^+/−^ (Reijns et al., 2012), suggesting that 50% RER activity of WT is sufficient to remove rNMPs from genomic DNA (Figure S2D). MEFs from *Rnaseh2a*^*G37S*/−^ embryos (on *p53*^+/+^ background) showed a distribution of DNA fragments similar to WT (Figure 4B) and contained about a third of the amount of rNMPs present in *Rnaseh2b*-null DNAs. Because *Rnaseh2a*^*G37S*/−^ embryos develop to birth, it suggests that 260,000 ± 40,000 rNMPs per cell, which is slightly but significantly less than the frequency found in *Rnaseh2a*^RED/G37S^, can be tolerated at least during embryogenesis.

As a complementary means to examine rNMPs in MEFs, we performed a single-cell gel electrophoresis (comet) assay under alkaline conditions, which detects both single- and double-strand breaks as well as alkaline-labile sites, including rNMPs in DNA. We used the same cell lines at the same cell passage, except for *Rnaseh2b*^−/−^, as for alkaline gel analysis. Greater than 90% of *Rnaseh2a^RED/RED^* MEF and *Rnaseh2b*-null cells’ DNAs migrated from the nucleus. With MEF cells from *Rnaseh2a*^+/+^ and *Rnaseh2a*^*RED*/+^ DNAs, in which little or no rNMPs are present, we found that only a small amount of DNA migrated from the nucleus (Figure 4D). Quantification of DNA damage by olive tail moment indicated that *Rnaseh2a*^*RED/RED*^ DNA is as seriously damaged as *Rnaseh2b*-null compared with *Rnaseh2a*^+/+^ and *Rnaseh2a*^*RED*/+^ (Figure 4D), consistent with alkaline gel results. Similarly, we observed elevated but significantly less DNA damage in *Rnaseh2a*^*G37S*/−^ and *Rnaseh2a^G37S/G37S^*. Importantly, we found that the comet assay of *Rnaseh2a^RED/G37S^* MEFs indicates a high level of rNMP retention but less than the *Rnaseh2a^RED/RED^* and *Rnaseh2b*-null cells. The results from alkaline gel and comet assays show clearly an inverse correlation between levels of rNMPs in DNA and embryonic progression.

## DISCUSSION

### Abundant Unrepaired rNMPs in DNA Produce Sufficient DNA Damage to Cause Early Embryonic Lethality

RNase H2 performs two distinct functions: hydrolysis of RNA/DNA hybrids formed during transcription and incision at rNMPs incorporated during DNA replication and repair. These two substrates are present simultaneously during the S and G2/M phases of the cell cycle and could compete for binding RNase H2. We are interested in establishing how these two activities contribute to cellular and organism fitness and viability. We have examined mice carrying a mutant form of RNase H2 (RNase H2^RED^) lacking RER of single rNMPs embedded in DNA and found that *Rnaseh2a^RED/RED^* embryos are similarly defective at the same stage of development as mice with null mutations in *Rnaseh2b* or *Rnaseh2c*. *Rnaseh2a^RED/RED^* MEFs retain about 65% of the null mutants’ unrepaired rNMPs in DNA, similarly inducing a p53 DNA damage response, which caused early embryonic arrest. We concluded that, as previously suggested (Hiller et al., 2012; Reijns et al., 2012), accumulation of rNMPs in DNA causes early embryonic lethality in null embryos, as well as in *Rnaseh2a*^RED/RED^.

### A Threshold of rNMPs in DNA for Survival of *Rnaseh2*-Defective Embryos

We found the abundances of rNMPs in DNA of *Rnaseh2a*^*G37S*/−^, *Rnaseh2a^RED/G37S^*, and *Rnaseh2a^RED/RED^* MEFs are approximately 27%, 37%, and 65%, respectively, of *Rnaseh2b*-null cells (Figure 4C). We believe that development to birth of *Rnaseh2a*^*G37S*/−^ pups having fewer, but still abundant, rNMPs indicates a threshold of ribonucleotides in DNA, above which the p53 damage response is activated, leading to early embryonic lethality. This order of relative abundance of rNMPs is confirmed by comet assay results (Figure 4D).

Support for this comes from our finding that we obtained MEFs from *Rnaseh2a^RED/RED^* embryos only in a p53^−/−^ background, not in a p53^+/+^ or p53^+/−^ background, whereas MEFs were readily obtained from *Rnaseh2a*^*G37S*/−^ embryos in a p53^+/+^ strain. We interpret these results to indicate that DNA damage in *Rnaseh2a*^−/−^, *Rnaseh2a^RED/RED^*, and *Rnaseh2a^RED/G37S^* MEF is so severe that p53 activation leads to irreversible genome instability and apoptotic death. *Rnaseh2a*^*G37S*/−^ and *Rnaseh2a^G37S/G37S^* embryos and MEFs do exhibit DNA damage but at a level that is tolerated, allowing their survival to birth (Figures 2 and 4). However, DNA damage in *Rnaseh2a*^*G37S*/−^ and *Rnaseh2a^G37S/G37S^* embryos activates an innate immune response (Pokatayev et al., 2016) (Figure 3B).

The fact that the absence of the p53 DNA damage response extends the viability of *Rnaseh2a^RED/RED^* embryos is an indication that dsDNA breaks may be the major contributor to early embryonic lethality. In yeast, topoisomerase 1 has been shown to generate breaks at rNMPs when RER is defective, and deletion of *Top1* allows viability in the presence of these increased rNMPs (Williams et al., 2013). Similarly, Top1-mediated nicks could induce DNA damage in mouse MEFs in the presence of rNMPs above the threshold. Replication-transcription collisions may also be contributing to dsDNA breaks. Perhaps different organisms have variable thresholds of ribonucleotides in DNA, or even different tissue and cell types could be more sensitive than others to rNMPs. Embryonic development, when DNA is being synthesized rapidly, seems to be a particularly susceptible stage. It would be interesting to determine whether after birth or in postmitotic tissues the RNase H2^G37S^ and or the RNase H2^RED^ mutants could become viable.

### RNase H2^RED^ Prohibits Completion of RER in the Presence of RNase H2^G37S^

The initial description of the RER pathway (Sparks et al., 2012) used purified WT *S. cerevisiae* enzymes in which the interaction between RNase H2 and the substrate did not require PCNA, thus describing the RER first step as incision of the rNMP by RNase H2. Our SPR data indicate that affinity of the RNase H2^RED^ and RNase H2^G37S^ enzymes for RER substrates is very low, and the competition experiments between the two mutant enzymes showed little or no interference for substrate binding (Figure S3A). We suggest that PCNA binding to either mutated enzyme aids in directing to or stabilizing the enzyme-1R-substrate interactions. In a *Rnaseh2a^RED/G37S^* strain, both mutant enzymes could compete for binding PCNA with only the RNase H2^G37S^:PCNA complex resulting in RER activity. RNase H2^RED^ might sequester PCNA or form stronger complexes, preventing RER. We hypothesize that an RNase H2^G37S^:PCNA interaction could promote RER, as demonstrated by the survival to birth of *Rnaseh2a*^*G37S*/−^ and *Rnaseh2a^G37S/G37S^* embryos (Figures 2 and S3C).

### First Structural Sign of Embryonic Defects Occurs at the Epiblast-to-Gastrulation Transition

Reijns et al. (2012) reported that differences in sizes of *Rnaseh2b*-null and WT embryos were first observed at E7.5. Between embryonic stage E6.5 and E7.5, cell numbers double every 5 hr, increasing 23-fold from 660 at E6.5 to 15,000 at E7.5 (Kojima et al., 2014). At this stage, almost 60% of the cell cycle is S phase, with short G1 and shorter G2/M phases. Embryos for *Rnaseh2a*^−/−^, *Rnaseh2a^RED/RED^*, and *Rnaseh2a^RED/G37S^* would be challenged to complete replication and repair of rNMPs in DNA in the context of epiblast expansion and gastrulation. Accumulation of rNMPs creates replication stress responses triggering a p53-dependent cascade of events that induced apoptosis and cell death, as indicated by much larger size of *Rnaseh2a^RED/RED^* embryos in a p53^−/−^ background.

Interestingly, we found that except for their smaller size, the mutant embryos of all RNase H2^G37S^ defective mice appear normal. At E10.5, WT embryos weigh about 10 mg, and at birth, pups weigh slightly more than 1 g. We estimate the weight of *Rnaseh2a^RED/G37S^* E10.5 embryos as ~1–2 mg (Figure 2A). At birth, the actual weight of perinatal-lethal *Rnaseh2a*^G37S/−^ pups approximates that of an E17 WT embryo (~900 mg) (Kojima et al., 2014; Macdowell et al., 1927) (Figure 2C). In the case of *Rnaseh2a*^*G37S*/−^ or *Rnaseh2a^G37S/G37S^* mice, the small size is observed as early as E10.5 and continues thereafter as if development were delayed by 1 day (Figure S2A), neither becoming relatively smaller nor able to catch up to normal size. We suggest that the smaller size results from fewer, yet normal, cells produced in the transition from epiblast to gastrulation.

### Are rNMPs in DNA the Cause of Perinatal Lethality in *Rnaseh^G37S^* Mice?

What, if any, is the contribution of R-loops to early embryonic death and to innate immune response induction? Formally, we cannot exclude the need for the hybrid activity of RNase H2 at any stage of development. Because the large numbers of rNMPs in DNA in *Rnaseh2a*-null, *Rnaseh2b*-null or *Rnaseh2c*-null, *Rnaseh2a^RED/RED^*, and *Rnaseh2a^RED/G37S^* stimulate the DNA damage response early in development, it may mask any defect due to hybrid accumulation. Does the perinatal lethality of *Rnaseh2a^G37S/G37S^* mice depend on the decreased abundance of rNMPs, or is there a role for Hybrid activity?

Our studies of functions of RNase H2 in *S. cerevisiae* showed that RNase H1 and H2 can share certain substrates, while only RNase H2 can recognize rNMPs. The synthetic sickness of *rnh201Δ sgs1Δ* strain requires the RNase H2 hybrid activity, which cannot be supplied by RNase H1 (Chon et al., 2013). Subsequent studies using the yeast RED mutation have permitted assignment of which of the two activities is relevant to alter a specific phenotype. For example, the Argueso lab (Conover et al., 2015; Cornelio et al., 2017) has shown in *S. cerevisiae* that both activities contribute to genome stability depending on the relative abundance of each substrate; when rNMPs are abundant, there is a greater requirement for RER activity, and when high frequencies of transcription of a particular sequence occur, there can be a larger need of the Hybrid activity. The Klein lab described accumulation of contiguous embedded rNMPs in the absence of RNase H2 that are RNA/DNA hybrids but not R-loops and are recognized by RNase H2 but not by RNase H1(Epshtein et al., 2016). In our *Rnaseh2a^G37S^* and *Rnaseh2a^RED^* mice, such differential requirement for the two activities could vary from tissue to tissue.

Recent reports demonstrate that defects in RNase H2 result in micronuclei that provide cytoplasmic DNA to stimulate the innate immune response via the cGAS-Sting pathway (Bartsch et al., 2017; Mackenzie et al., 2017). DNA damage leading to cytoplasmic DNA could occur in RNase H2-defective mice and humans either via dsDNA breaks, as seen in E2-stimulated estrogen transcription induced R-loops (Stork et al., 2016), or resulting from rNMPs in DNA (Cerritelli and Crouch, 2016; Epshtein et al., 2016; Williams et al., 2016). Radiotherapy and chemotherapeutics can cause DNA strand breaks and formation of micronuclei, which also activate the cGAS-Sting pathway (Harding et al., 2017).

Both RNA/DNA hybrids and rNMPs in DNA are known to cause dsDNA breaks, and each could activate the cGAS-Sting DNA immune-activating pathway or otherwise induce p53-dependent DNA damage response. The RNase H2^RED^ enzyme both *in vitro* and *in vivo* has no significant activity cleaving at rNMPs in DNA. Recent studies in human tissue culture cells have exploited the differential activities of RNase H2^RED^ to assign which of its two activities is required for (1) retrotransposition of Line-1 (Benitez-Guijarro et al., 2018) and (2) providing sites in DNA for interaction with PARP1 (Zimmermann et al., 2018). The former needs RNA/DNA-resolving activity and the latter the retention of rNMPs in DNA. These cultured cells can survive without RNase H2, unlike our *Rnaseh2a^RED/RED^* mice, which arrest during early embryonic development. It will be interesting to see the properties of specific tissues when RNase H2^RED^ is the only RNase H2 present. A mutant defective in Hybrid hydrolysis but fully capable of RER activity may reveal which nucleic acid is responsible for the autoimmune response found in *Rnaseh2a*^*G37S/*G37S^ mice and ultimately in AGS patients with the G37S mutation as well as aid in defining roles for RNA/DNA hybrids that are exclusively recognized by RNase H2.

## STAR★METHODS

### KEY RESOURCES TABLE

**Table:** 

REAGENT or RESOURCE	SOURCE	IDENTIFIER
Antibodies		
Rabbit polyclonal anti-beta actin	Abcam	ab8227; RRID:AB_2305186
Rabbit polyclonal anti-RNase H2 subunit A	Proteintech	Cat#16132-1-AP; RRID:AB_2269729
Rabbit polyclonal anti-RNase H2 subunit C	Abcam	ab89726; RRID:AB_2042815
Goat polyclonal anti-rabbit IgG	Abcam	ab205718
Bacterial and Virus Strains		
One Shot BL21(DE3) competent cells	Invitrogen	C600003
One Shot MAX Efficiency DH5α-T1R competent cells	Invitrogen	Cat#12297016
Chemicals, Peptides, and Recombinant Proteins		
Mouse RNase H2	This study	N/A
*E. coli* RNase HII	New England Biolabs	M0288S
T4 Polynucleotide Kinase	New England Biolabs	M0201S
Phosphodiesterase I	Worthington Biochemical	LS003926
Streptavidin	Sigma-Aldrich	S4762
Pierce Bovine Serum Albumin Standard’	Thermo Scientific	Cat#23209
HBS-EP Buffer	GE Healthcare	BR-1001-88
Newborn Calf Serum	GIBCO	Cat#16010-159
DMEM	GIBCO	Cat#12430-054
L-Glutamate	GIBCO	Cat#25030-081
Penicillin Streptomycin	GIBCO	Cat#15140-148
DPBS	GIBCO	Cat#14190144
10×Tris/Glycine/SDS running buffer	BIORAD	Cat#161-0732
Blotting-Grade Blocker	BIORAD	Cat#1706404
PureLink DNase	Invitrogen	Cat#12185-010
LightCycler 480 SYBR Green I Master	Roche	Cat#04707516001
CometSlides	Trevigen	Cat#4250-200-03
Pierce ECL Western Blotting Substrate	Thermo Scientific	Cat#32106
Critical Commercial Assays		
Quikchange II site-directed mutagenesis kit	Agilent technologies	Cat#200524
Histrap crude FF columns	GE Healthcare	Cat#11-0004-58
Hitrap Heparin HP columns	GE Healthcare	Cat#17-0406-01
HiLoad 16/600 Superdex 200 pg columns	GE Healthcare	Cat#28989335
Quick-DNA Universal kit	Zymo Research	D4068
QIAprep Spin Miniprep kit	QIAGEN	Cat#27106
DNeasy Blood and Tissue kit	QIAGEN	Cat#69506
Trizol	Life technologies	Cat#15596018
PureLink RNA Mini kit	Invitrogen	Cat#12183018A
Omniscript RT Kit	QIAGEN	Cat#205113
Comet assay kit	Trevigen	Cat#4250-050-K
Experimental Models: Cell Lines		
Mouse: embryonic primary fibroblasts	This study	N/A
Mouse: ES Cells *Rnaseh2a*^−/−^	KOMP	*_Rnaseh2a_ tm1(KOMP)Wtsi*
Mouse: *Rnaseh2b*-null cell line	Rejins and Jackson	N/A
Mouse: *Rnaseh2a*^−/−^	This study	N/A
Mouse: *Rnaseh2a*^*G37S*/−^	This study and Pokatayev et al., 2016	N/A
Mouse: Sting−/−	KOMP	*_Tmem173_ tm1.1(KOMP)Mbp*
Mouse: p53	Jackson Lab Mice	B6.129S2-*Trp53* ^tm1Tyj^/J
Oligonucleotides		
Primers for genotyping, see Table S1	This study	N/A
Primers for RT-qPCR, see Table S1	This study	N/A
RNase H2 substrate oligos, see Table S1	This study	N/A
Short hairpin DNA for Biacore, see Table S1 and Figure S6	This study	N/A
Oligo(dT)18 Primer	Thermo Scientific	SO131
Recombinant DNA		
Plasmid: pET15b	Novagen	S4762
Software and Algorithms		
ImageJ	NIH	https://imagej.nih.gov/ij/
Opencomet plugin	Gyori et al., 2014	http://www.cometbio.org/index.html
Math Works SLM tool	NIH	http://www.mathworks.com/matlabcentral/fileexchange/24443

### CONTACT FOR REAGENT AND RESOURCE SHARING

Further information and requests for resources and reagents should be directed to and will be fulfilled by the Lead Contact, Robert J. Crouch (crouchr@mail.nih.gov).

### EXPERIMENTAL MODEL AND SUBJECT DETAILS

#### Mice

Mice and cell lines used in this study are listed in Key Resources Table. *Rnaseh2aKO*, STING−/−, *RnasehG37S* and *Rnaseh2aRED* were all originally produced in C57BL6/N ES cells. B6.129S2-Trp53tm1Tyj/J mice 6J. Mice for removal of drug-resistant markers employed were also C57BL/6 but if 6N or 6J not known. Thus, the mice studied in this investigation most likely contain mixtures of 6N and 6J genomes but with a strong bias for 6N genes.

All animal care and experimental procedures were performed in accordance with the ethical guidelines approved by NIH’s Office of Animal Care and Use 15-029 (OACU). The day when we confirmed vaginal plugs was defined as E0.5, and the day of birth was as P0. Primary MEF cell lines were prepared from E10.5 or E14.5 embryos of either sex and cultured in DMEM at 37°C in humidified incubator under 5% CO_2_.

*Rnaseh2a^RED^* (P40D Y211A) ES cells were first made for the P40D mutation by modification by Quikchange of the plasmid used to produce the *Rnaseha^G37S^* mouse (Pokatayev et al.,2016) by neomycin selection. These ES cells were then used to introduce the Y211A mutation by hygromycin selection. Each cassette for drug selection contained an I-Scel site. To ensure the mutations resided on the same allele, DNA from neomycin-hygromycin selected cells was digested by I-Scel and a ~10kbp DNA fragment was detected by Southern analysis resistance genes were used for positive selection of double mutation and subsequently deleted from knock-in mice by Cre- and Flip-dependent recombination. *Rnaseh2a^G37S^* knock-in mice were as described previously (Pokatayev et al., 2016) except the region between the loxP site was removed. *Rnaseh2a^tm1(KOMP)Wtsi^* ES cells and *Tmem173^tm1.1(KOMP)Mbp^* (Sting) KO mice were obtained from the Knockout Mouse Project (KOMP) repository. *Rnaseh2a^tm1(KOMP)Wtsi^* ES cells were used to generate heterozygous *Rnaseh2a*^+/−^. B6.129S2-*Trp53*^tm1Tyi^/J (p53) mice were obtained from The Jackson Laboratory. *Rnaseh2a*KO, STING−/−, *RnasehG37S* and *Rnaseh2a*RED were all originally produced in C57BL6/N ES cells. B6.129S2-Trp53tm1Tyj/J mice are 6J. Mice for removal of drug-resistant markers employed were also C57BL/6 but if 6N or 6J not known. Thus, the mice studied in this investigation most likely contain mixtures of 6N and 6J genomes but with a strong bias for 6N genes. Sex of embryos were not determined. All mouse studies were approved by the NICHD Animal Use Committee protocol 15-029.

### METHODS DETAILS

#### Genotyping

For mouse genotyping, DNA was isolated from mouse tail-clips or embryo yolk sacs with DNeasy Blood & Tissue kit (QIAGEN) according to manufacturer’s instructions. Genotypes were verified by PCR with *Taq* polymerase (Roche).

#### Generation of MEFs

For generation of *Rnaseh2a^RED/RED^ p53*^−/−^, *Rnaseh2a^RED/G37S^ p53*^−/−^, *Rnaseh2a*^*RED*/+^ *p53*^−/−^ and *Rnaseh2a*^+/+^ *p53*^−/−^ MEFs, whole embryos at E10.5 were minced and cultured in DMEM with 10% FBS, 2 mM L-Glutamine, 100 U/ml penicillin, 100 μg/ml streptomycin (GIBCO), 0.1 mM β-Mercaptoethanol, initially in 12-well plate at 37°C, 5% CO_2_. *Rnaseh2a*^*G37S*/−^ *p53*^+/+^ and *Rnaseh2a*^+/+^ *p53*^+/+^ MEFs were also established using E14.5 embryos as described above after removing head, limbs, tail and red organs from the embryos. After incubation for 24 h, culture medium was exchanged with fresh medium to remove non-adherent cells. Adherent cells were subsequently maintained and passaged as appropriate under the same condition.

#### Western blotting

Harvested MEFs were washed twice in ice-cold PBS, resuspended in buffer containing 50 mM Tris-HCl (pH 8.0), 60 mM KCl, 0.1% Triton X-100 and 100-fold diluted protease inhibitor cocktail (Calbiochem) and disrupted by sonication. Insoluble proteins were removed by centrifugation. The concentration of soluble proteins was determined with Pierce BCA Protein Assay kit (Thermo Scientific) using bovine serum albumin as a standard. Equal amount of protein homogenates was loaded onto 10%–20% SDS-PAGE gel and separated. Upon electrophoresis, gel was soaked with 20% ethanol for 5 min and proteins were transferred to PVDF membrane with iBlot 2 Dry Blotting System (Invitrogen). Membrane was blocked with 5% milk (BIORAD) in TBST (50 mM Tris-HCl pH7.5,150 mM NaCl, 0.2% Triton X-100) at RT for 1 h and subsequently incubated with diluted antibodies (1:1000 for anti-RNase H2A antibody (Proteintech) or 1:2000 for anti-RNase H2C and anti-β-actin antibodies (Abcam)) in TBST containing 0.5% milk at RT for 1 h with agitation. After three washes with TBST, the membrane was incubated with 20000-fold diluted anti-rabbit IgG secondary antibody (Abcam) in TBST containing 0.5% milk at RT for 1 h and further washed three times with TBST plus 0.5% milk. HRP activity from antibody was detected by soaking membrane with Pierce ECL western blotting substrate (Thermo Scientific) at RT for 1 min and subsequently scanning chemiluminescent signal on membrane with Azure C600 (Azure Biosystem).

#### Preparation of recombinant mouse RNase H2

Three genes encoding mouse RNase H2 subunits (*Rnaseh2a, Rnaseh2b* and *Rnaseh2c*) were cloned from cDNA and inserted into pET15b expression vector as a polycistronic construct as was done for expression of human RNase H2 (Chon et al., 2009). All proteins bear the N-terminal His tag removable with thrombin. The pET15b derivatives for overproduction of RNase H2^RED^ and RNase H2^G37S^ were constructed with Quikchange mutagenesis kit (Stratagene) by introducing point mutations into *Rnaseh2a*.

Mouse RNase H2 was overproduced in *E. coli* BL21(DE3) and purified as described for human RNase H2 (Chon et al., 2009). When further purification is required, the protein was loaded to Hiload 16/60 Superdex 75pg column (GE Healthcare) equilibrated with 20 mM Tris-HCl (pH 7.5) containing 1 mM EDTA and 150 mM NaCl and the fractions containing 1:1:1 complex of three RNase H2 subunits were collected. The purity of proteins was confirmed by 10%–20% gradient SDS-PAGE (Bio-Rad), followed by Coomassie Brilliant Blue staining. Protein concentrations were determined by measuring the absorption at 280 nm (A280) and the A280 values for 0.1% (1.0 mg/ml) protein solution are 0.85 for RNase H2 and RNase H2^G37S^ and 0.83 for RNase H2^RED^. These values were calculated by using absorption coefficient of 1576 M^−1^ cm^−1^ for Tyr and 5225 M^−1^ cm^−1^ for Trp at 280 nm (Goodwin and Morton, 1946).

#### RNase H activity assays

We employed ^32^P-labeled substrates because we found the fluorescent substrates previously used (Crow et al., 2006; Reijns et al., 2012) were less well hydrolyzed by RNase H2^RED^. When we used the same 18 bp hybrid substrates labeled with ^32^P-5′-RNA or with the fluorescent label, we found RNase H2^RED^ to be 20% as active as RNase H2^WT^ by autoradiography, compared to ~10% with autofluorography (Figure S1B). When we measured by fluorescence emission, the activity of RNase H2^RED^ was even lower (~4%) (Figure S1B). Interestingly, RNase H2^RED^ failed to cleave at several sites, one of them near the 3′ end with the FAM tag. This modest change in site selection was independent of the FAM moiety and explains the difference in product sizes seen by autofluorography (Figure S1B). Two recent reports have made similar observations for human RNase H2^RED^, including reduced affinity toward the same FAM-modified 18R substrate (Benitez-Guijarro et al., 2018; Zimmermann et al., 2018). Taken together, these data indicate that RNase H2^RED^ activity is compromised toward short RNA/DNA duplexes, but it is proficient in removing long RNA/DNA hybrids, which are presumably present in R-loops.

Enzyme activity assays were performed using 5′-^32^P-labeled 12 base DNA_5_-RNA_1_-DNA_6_ (5′-GACACcTGATTC-3′) hybridized with 2 molar equivalents of the complementary DNA. Other 5′-^32^P-labeled RNAs were similarly treated. Uniformly labeled poly-^32^P-rA/poly-dT to determine Hybrid activity was synthesized as described (Crouch et al., 2001). The reaction buffer was 50 mM Tris-HCl (pH 8.0) containing 50 mM NaCl, 10 mM MgCl_2_,1 mM dithiothreitol, 100 μg/ml BSA and 5% (w/v) glycerol. Products of 1 μM substrates were applied to a 20% or 12% TBE-urea polyacrylamide gel and quantified by Typhoon imager upon electrophoresis.

For preparation of embryo homogenates, whole embryos at E11.5 were washed twice in ice-cold PBS and sonicated on ice in the buffer containing 50 mM Tris-HCl (pH 8.0), 60 mM KCl, 0.1% Triton X-100 and 100-fold diluted protease inhibitor cocktail. The supernatant was collected by centrifuge and used for activity assays. The concentration of total proteins was determined with Pierce BCA Protein Assay kit as described for western blot (see above).

#### Alkaline gel analysis of rNMPs in DNA

Genomic DNA was isolated from MEFs using Quick DNA Universal kit (Zymo Research). For alkaline hydrolysis, 1 μg of the genomic DNA was incubated with 0.3 M NaOH at 55°C for 2 h. After alkaline treatment, 6 × loading buffer (300 mM NaOH, 6 mM EDTA, 18% (w/v) Ficoll PM400 and Orange G) was added to treated DNA samples. 0.7% agarose gel was equilibrated by incubating in running buffer (50 mM NaOH and 1 mM EDTA) for 1 h. Electrophoresis was performed at 1 V cm^−1^ for 18 h. Upon electrophoresis, the gel was neutralized by soaking with 0.7 M Tris-HCl (pH 8.0) containing 1.5 M NaCl and stained with SYBR Gold. Gel images were obtained using UVP BioSpectrum AC Imaging System. Densitometric quantification of DNA in gel was performed with ImageQuant TL software (GE Healthcare).

#### Comet assay

The comet assay was performed with Comet Assay Electrophoresis System (Trevigen) following manufacturer’s instructions with some modifications. MEFs were synchronized by serum starvation for 24 h in serum-free DMEM medium and subsequently harvested, washed and suspended in ice-cold PBS at 2.0 × 10^5^ cells/ml. LMAgarose (Trevigen) incubated at 37°C was added to suspended cells at a ratio of 10:1 (v/v) and the mixture was dispersed on comet slides (Trevigen). After gelling at 4°C, slides were soaked with 10 mM Tris-HCl (pH 9.5) containing 2.5 M NaCl, 0.1 M EDTA and 1% (v/v) Triton X-100 at 4°C for 16–18 h. Slides were then incubated in running buffer (0.2 M NaOH, 1 mM EDTA) for 20 min at room temperature. Electrophoresis was performed at 21 V for 15 min. Upon electrophoresis, slides were washed twice in dH_2_O and 70% ethanol and dried at 37°C. The DNA was stained with SYBR-Gold for 30 min at room temperature and briefly rinsed with dH_2_O. Comet images were captured with epifluorescence microscopy. Olive tail moments were determined by analyzing at least 100 comets/slide with OpenComet software (Gyori et al., 2014).

#### RT-qPCR

For RNA isolation, mouse embryos were washed in ice-cold PBS and homogenized in Trizol reagent (Invitrogen). Upon addition of chloroform, total RNA was isolated from water phase and purified with PureLink RNA Mini Kit columns (Ambion) and PreLink DNase (Invitrogen). cDNA was reverse transcribed from isolated RNA with Omniscript RT kit (QIAGEN) using oligo-dT primers (Thermo Scientific). Quantitative PCR was performed with SYBR Green I Master reagent (Roche) using Lightcycler 480 system (Roche). The expression of target genes was calculated by E-method (Tellmann, 2006) and normalized to the housekeeping gene (β-actin). All primer sequences are listed in Table S1.

#### Biosensor analysis

The binding properties of mouse RNase H2 to single ribonucleotide in DNA duplex were examined by surface plasmon resonance (SPR) analysis using Biacore 3000 instrument (GE Healthcare Life Science). All experiments were performed at 25°C in triplicate and repeated on three different sensor chips. The running buffer was HBS (10 mM HEPES pH 7.4, 150 mM NaCl, 3 mM EDTA and 0.005% Surfactant P20). A hairpin ligand DNA containing a single ribonucleotide in the stem of the hybrid and a biotinylated T in the loop (Figure S3A) (Gaidamakov et al., 2005) was immobilized on a streptavidin-coated CM4 sensor chip through biotin-streptavidin interaction. CM4 sensor chips were preconditioned by two times alternating injections of 10 mM HCl, 50 mM NaOH and 0.1% (w/v) SDS and then washed with water. Streptavidin was immobilized using amine coupling method by injection of 0.1 mg/ml streptavidin in HBS for 5 min. The surface was activated by injecting 1:1 mixture of 0.4 M 1-ethyl-3-(3-dimethylamino-propyl)-carbodiimide and 0.1 M N-hydroxysuccinimide at 5 μl/min of flow rate for 7 min, followed by injection of 0.01 mg/ml streptavidin in 10 mM sodium acetate (pH 5.0) for 7 min followed by 1 M ethanolamine hydrochloride for 7 min to deactivate excess reactive groups. Non-covalently bound streptavidin was eliminated by four alternate injections of 100 μL of 1 M NaCl and 50 mM NaOH at 50 μl/min. The single-stranded loop ligand DNA was diluted to 2 nM in HBS, heated at 90°C for 1 min and immediately transferred on ice. Then, ligand DNA was injected at 10 μl/min until about 40-50 RU of ligand was captured on the surface. Proteins were serially diluted at final concentrations in HBS. To minimize mass transport effects, proteins were injected at fast flow rate 60 μl/min for 90 s and then dissociation was monitored for 5 min. Surface regeneration was performed by duplicate injections of 20 μL of 10 mM HCl. To avoid protein contamination on surface, the binding of one protein was continuously measured at different concentrations and the surface was washed with 20 μL of 50 mM NaOH before injection of another protein. Data processing and analysis was performed using BIAevaluation 4.1 software (BIACORE).

### QUANTIFICATION AND STATISTICAL ANALYSIS

All statistical details in experiments were described in figures and figure legends. Comet assay data was analyzed by Student’s t test using Microsoft Excel. Also see above for the details of quantification of ribonucleotides in genomic DNA.

Ribonucleotide incorporation abundance were estimated through a slightly modified version of the method described in (Reijns et al., 2012). Starting from the densitometric histograms of electrophoresis lanes corresponding to alkali-treated genomic DNA, the background intensity was subtracted and a smoothing spline with 15 optimally placed internal knots was applied to the densitometric profiles by running the SLM tool (D’Errico, August 10, 2017(http://www.mathworks.com/matlabcentral/fileexchange/24443)) in MATLAB version 9.2. The smoothened intensity curves were resampled at intervals of width Δ*d* = 1mm. For each interval, the characteristic fragment size (*sz*) was calculated following the equation *d* = *a* + *b* × log(*sz*), where *d* represents the electrophoretic distance in the middle point of the interval. Parameters *a* and *b* were inferred by fitting the linear model *lm*(*d* ~ log (*sz*)) to the peaks in the size reference lane. Because the conversion from densitometric intensity to fragment count is highly sensitive to small, noisy fluctuations in the far end of the electrophoretic gel, a cutoff at an electrophoretic distance *d_max_* was introduced. The value of *d_max_* was determined under supervision, as the point where fluctuations in the original (non-smoothened) intensities of the loaded lanes became similar in magnitude to those observed in empty lanes, indicating a poor signal-to-noise ratio. The fragment count associated to each interval was estimated as *n_sz_* = *I_sz_/sz*, being *I_sz_* the densitometric intensity in the interval. From these numbers, a preliminary estimate of the mean fragment size was obtained as $\bar{\mathrm{sz}}=\Sigma (\mathrm{sz}\;n_{\mathrm{sz}})∕\Sigma n_{\mathrm{sz}}$, where the sum extends over all the intervals. To account for small fragments that had migrated beyond *d_max_*, the mean fragment size was corrected under the assumption that break points are randomly distributed along the genome. Thus, the corrected estimate for the mean fragment size became ${\bar{\mathrm{sz}}}_{\mathrm{corr}}=\bar{\mathrm{sz}}\times \exp(-{\mathrm{sz}}_{\min}∕\bar{\mathrm{sz}})$, where *sz_min_* is the minimum size cutoff corresponding to *d_max_*.

Given a genome of size *G*, the number of breakpoints is readily obtained as $N=G∕{\bar{\mathrm{sz}}}_{\mathrm{corr}}$. Using the size of the mouse genome as reference, the number of break points was calculated for every sample. The excess of break points associated to a mutation was obtained by subtracting the number of break points in the WT to the number of break points in the mutant. Finally, the increase in the ribonucleotide incorporation rate associated to the mutation was computed as (N_WT – N_mutant)/G.

## Supplementary Material

1

## Highlights

- Excess ribonucleotides in DNA cause mouse early embryonic death
- Above a threshold of tolerance, ribonucleotides in DNA induce apoptotic cell death
- Below the threshold, ribonucleotides in DNA trigger an innate immune response
- Competition between RNases H2 mutants affects rNMP removal and cell survival
