# Supplementary material for: Two RNase H2 Mutants with Differential rNMP Processing Activity Reveal a Threshold of Ribonucleotide Tolerance for Embryonic Development
Source: Cell Rep. Author manuscript; Available in PMC 2018 Dec 28. (PMC6309994; doi:10.1016/j.celrep.2018.10.019)
Supplement: 1 [file NIHMS1512005-supplement-1.pdf]

Cell Reports, Volume *25*

Supplemental Information

Two RNase H2 Mutants with Differential rNMP Processing Activity Reveal a Threshold of Ribonucleotide Tolerance for Embryonic Development

Ryo Uehara, Susana M. Cerritelli, Naushaba Hasin, Kiran Sakhuja, Mariya London, Jaime Iranzo, Hyongi Chon, Alexander Grinberg, and Robert J. Crouch

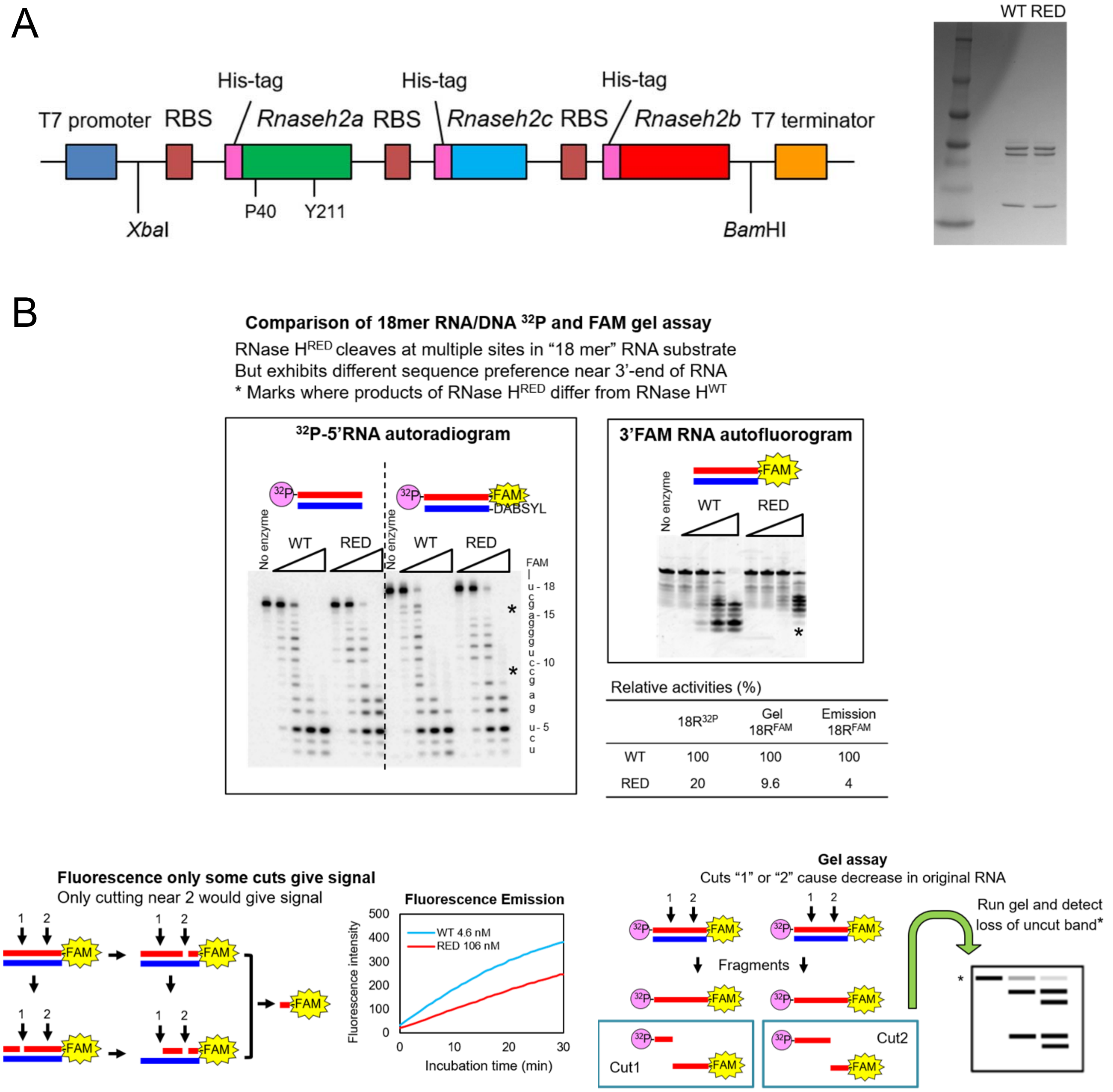

	$18R^{32P}$	Gel $18R^{FAM}$	Emission $18R^{FAM}$
WT	100	100	100
RED	20	9.6	4

Figure S1. In vitro characterization of RNase H2^{RED}. Related to Figure 1 and STAR Methods. (A) Expression vector and purified protein. *Rnaseh2a*, *Rnaseh2c* and *Rnaseh2b* cDNAs were cloned and fused with N-terminal His-tag on pET15b vector as shown above. RED mutation (P40D and Y211A) was introduced by Quikchange. Three subunits of mouse RNase H2^{WT} and RNase H2^{RED} were expressed in BL21(DE3) *E. coli* strain, purified as described in STAR Methods, and analyzed by 10-20% SDS-PAGE as shown below. (B) Comparison of ^{32}P-labeled and fluorescent substrate assays. Top; RNaseH2^{RED} fails to cleave several sites of 18bp RNA/DNA hybrids. Autoradiogram of ^{32}P-labeled substrates (left) in sequencing acrylamide gel and autofluorogram (right) of the same substrate upon enzyme cleavages. Relative activities are shown in table when measured by loss of starting substrate. An activity toward 18R-FAM was determined by both gel assay and emission measurement. Enzymes are RNase H^{WT} (WT) and RNaseH2^{RED} (RED). Bottom; Cleavages by RNase H2^{RED} may not be efficient to release FAM modified RNA from DNA (left). Cartoon of assays to detect fluorescence emission from FAM modified substrate upon cleavage by RNase H2. RNase H2^{RED} activity was determined to be about 4% of wild type from fluorescence emission curves. Cartoon of each assay and that ^{32}P assay detects all multiple cleavages (right).

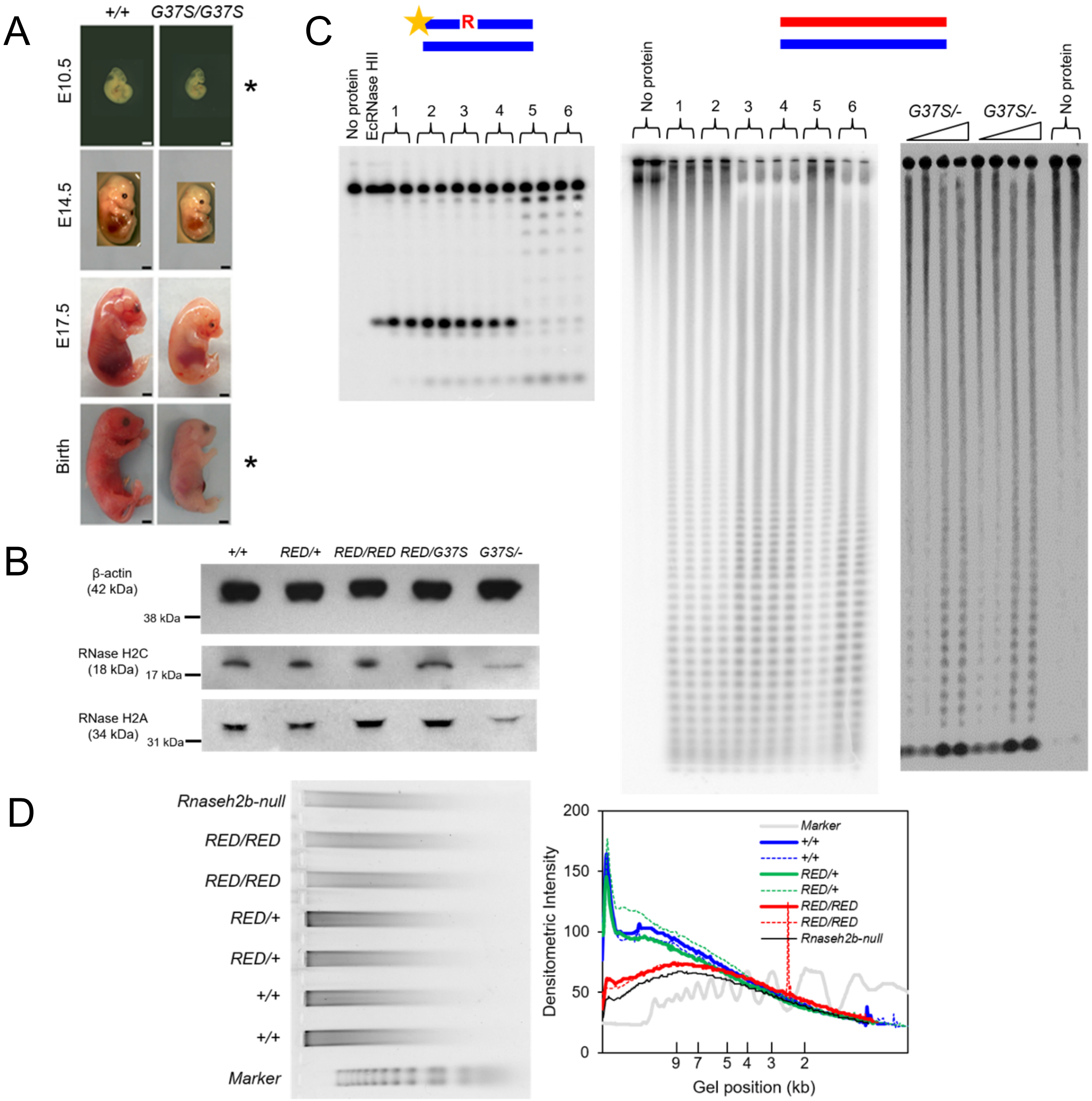

Figure S2. Loss of RER activity leads to embryonic lethality in mouse. Related to Figure 2 and 4. A) Perinatal lethality of AGS-hypomorphic mutant mice. *Rnaseh2a*$^{+/+}$ (+/+) and *Rnaseh2a*$^{G37S/G37S}$ (G37S/G37S) embryos and neonates. Scale bar, 1 mm. Mice pictures at E10.5 and at birth (marked with *) are same as paper previously published (Pokatayev, J. Exp. Med. 2016). B) Western blot using MEF homogenates. Protein lysates from primary MEFs *Rnaseh2a*$^{+/+}$ *p53*$^{-/-}$ (+/+), *Rnaseh2a*$^{RED/+}$ *p53*$^{-/-}$ (RED/+), *Rnaseh2a*$^{RED/RED}$ *p53*$^{-/-}$ (RED/RED), *Rnaseh2a*$^{RED/G37S}$ *p53*$^{-/-}$ (RED/G37S), *Rnaseh2a*$^{G37S/-}$ *p53*$^{+/+}$ (G37S/-) were separated by 10-20% SDS-PAGE, transferred to PVDF membrane and probed with anti-β-actin antibodies (Abcam), anti-RNase H2C (Abcam) and anti-RNase H2A (Proteintech). C) Cleavage of 1R and poly-rA/poly-dT substrates with embryo lysates. Embryos lysates were prepared as described in Experimental Procedures and used for assays. Enzyme reaction was performed at 37˚C for 30 min in duplicate. Reaction products were analyzed by (left) 20 and (middle, right) 12% TBE-Urea PAGE. (Left and middle) Lane 1 and 2, two *Rnaseh2a*$^{+/+}$ embryos, lane 3 and 4, *Rnaseh2a*$^{RED/+}$ embryos, lane 5 and 6, *Rnaseh2a*$^{RED/RED}$ embryos. (Right) Two *Rnaseh2a*$^{G37S/-}$ (G/-) embryos. No protein lane contained homogenization buffer and EcRNase HII lane contained 10 U of *E. coli* RNase HII instead of embryo lysates. The two gel pictures on the right were separately uniformly modified in pptX file using Format "artist". D) No significant rNMPs accumulation in *Rnaseh2a*$^{RED/+}$ DNA. Genomic DNA was isolated from cell lines established from two independent *Rnaseh2a*$^{+/+}$ *p53*$^{-/-}$, *Rnaseh2a*$^{RED/+}$ *p53*$^{-/-}$, *Rnaseh2a*$^{RED/RED}$ *p53*$^{-/-}$ embryos and analyzed by alkaline agarose gel electrophoresis with molecular marker and control (*Rnaseh2b-null*). The genotype of each cell line is shown in the inset of the figure.

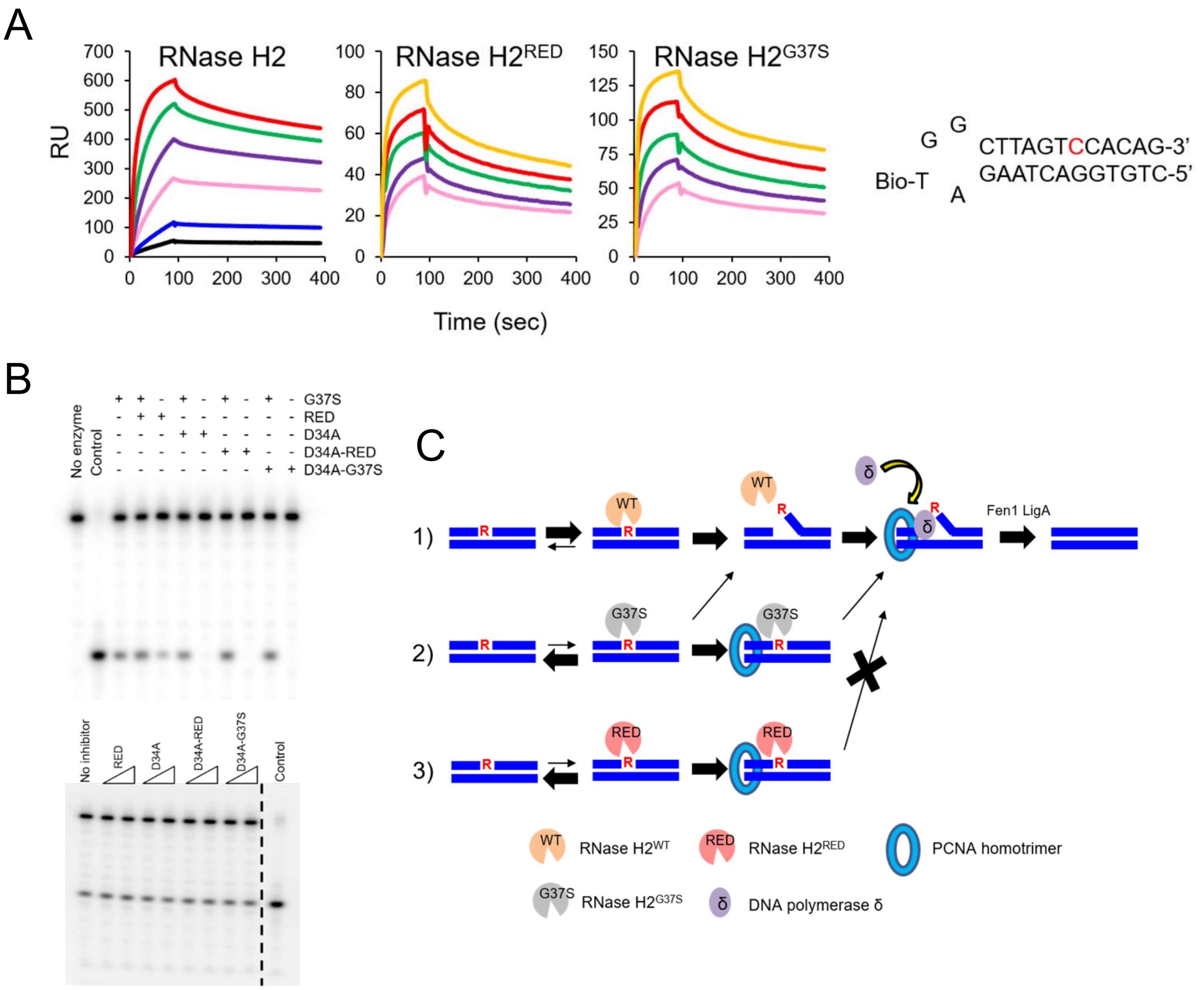

Figure S3. Competition of RNase H2^{RED} and RNase H2^{G37S} for removal of rNMP. Related to Figure 2 and 4, and STAR Mthods. A) Surface plasmon resonance binding curves of RNase H2, RNase H2^{RED} and RNase H2^{G37S} to single ribonucleotide embedded in 12 bp hairpin DNA duplex immobilized on the sensor chip. The name of protein analyte is indicated above each set of curves. Immobilized DNA sequence is shown on the right of SPR curves. Single ribonucleotide (rC) is colored red. Bio-T indicates biotin modified dT on which DNA is attached to the streptavidin immobilized on the censor chip. Increasing concentrations of protein solutions were injected for 90 sec over the surface and subsequently monitored for 300 sec. Each curve represents 5 nM (black), 10 nM (blue), 25 nM (pink), 50 nM (purple), 100 nM (green), 200 nM (red) and 400 nM (orange) protein loading. B) Degradation of 1R substrate by RNase H2^{G37S} in the presence of inactive mutants. Top; An activity of 20 nM RNase H2^{G37S} in the presence of 2000 nM inhibitors (RED and inactive D34A mutants) was determined using 20 nM 1R substrate. All components except $MgCl_2$ were pre-mixed and incubated for 3 min at RT. Enzyme reaction was initiated by addition of $MgCl_2$ and performed for 1 min at RT. Control lane contained an equivalent amount of RNase H2^{WT} without inhibitors. Relative activities were determined by estimating the amounts of cleaved products. The experiment was performed in triplicate. RNase H2^{RED} and three inactive mutants did not affect cleavage of 1R substrate, suggesting that these mutants do not compete with RNase H2^{G37S} for binding to single rNMP in dsDNA. Bottom; Incision of rNMP in dsDNA by RNase H2^{G37S} was independent on the concentration of inhibitors. An activity of RNase H2^{G37S} was determined in the presence of 10- or 100-fold inhibitors. C) Schematic model of competition of two defective mutants in RER pathway. Each RNase H2^{RED} and RNase H2^{G37S} poorly bind rNMPs in DNA. Only RNase H2^{G37S} proceeds to repair synthesis. Association with rNMPs and recruitment of PCNA can be competitive between two mutants. Canonical RER pathway (1) does not require PIP of RNase H2. Binding of RNase H2^{G37S} (2) and RNase H2^{RED} (3) is facilitated by PCNA leading to an inactive complex for RNase H2^{RED}, which limits access of RNase H2^{G37S} to rNMPs in DNA. Note arrow weight reflects preferred direction.

Table S1. Oligo sequences used in this study. Related to STAR Methods.

Name	Sequence (5' to 3')	Purpose	Source
Rnaseh2a-P40D fwd	GGTCAGCATCAAGAGCCACTGCAG	Genotyping	Lofstrand Labs
Rnaseh2a-P40D rev	CCCGCTCGTTCTCTGTCAAGG	Genotyping	Lofstrand Labs
Rnaseh2a-Y211A fwd	TCCCGGGATAGAGGTGACAG	Genotyping	Lofstrand Labs
Rnaseh2a-Y211A rev	AAGCTGGAGACCAGCCTGGG	Genotyping	Lofstrand Labs
Rnaseh2a-del fwd	GAGACCAGTGTTCCGCCTGTGC	Genotyping	Lofstrand Labs
Rnaseh2a-del rev	CAGGCTTCACTGAGTCTCTGGC	Genotyping	Lofstrand Labs
p53-ko fwd	CAGCCTCTGTTCCACATACACT	Genotyping	Lofstrand Labs
p53-ko rev	TGGATGGTGGTATACTCAGAGC	Genotyping	Lofstrand Labs
Sting-ko fwd	GGCGGTGATTTTGGCGATAC	Genotyping	Lofstrand Labs
Sting-ko rev	AATGCGGGTCGCTTCACTTA	Genotyping	Lofstrand Labs
Actin fwd	CTAAGGCCAACCGTGAAAAG	Quantitative PCR	Lofstrand Labs
Actin rev	ACCAGAGGCATACAGGGACA	Quantitative PCR	Lofstrand Labs
Rnaseh2a fwd	GGTCGTTGTCGTCTGAGTTC	Quantitative PCR	Lofstrand Labs
Rnaseh2a rev	AGATCTGCCAAGCGAGACAG	Quantitative PCR	Lofstrand Labs
Cdkn1a fwd	TCCACAGCGATATCCAGACA	Quantitative PCR	Lofstrand Labs
Cdkn1a rev	GGACATCACCAGATTGGAC	Quantitative PCR	Lofstrand Labs
Ccng1 fwd	TTAGTAGGCCTGTCGGATCG	Quantitative PCR	Lofstrand Labs
Ccng1 rev	AGCAGTTTCTGAGAGTCAGTTGTC	Quantitative PCR	Lofstrand Labs
Ifit1 fwd	TCTAAACAGGGCCTTGCAG	Quantitative PCR	Lofstrand Labs
Ifit1 rev	GCAGAGCCCTTTTTGATAATGT	Quantitative PCR	Lofstrand Labs
Ifit3 fwd	TGAACTGCTCAGCCCACA	Quantitative PCR	Lofstrand Labs
Ifit3 rev	TCCCGGTTGACCTCACTC	Quantitative PCR	Lofstrand Labs
Cxcl10 fwd	ATGACGGGCCAGTGAGAATG	Quantitative PCR	Lofstrand Labs
Cxcl10 rev	ATTCCGGATTCAGACATCTCT	Quantitative PCR	Lofstrand Labs
1R substrate	GACACcTGATTC	Enzyme activity	IDT
12R substrate	gacaccugauuc	Enzyme activity	IDT
12 mer reverse complement	GAATCAGGTGTC	Enzyme activity	Lofstrand Labs
1R substrate (fluorescence)	GATCTGAGCCTGGGaGCT	Enzyme activity	TriLink
18R substrate (fluorescence)	gaucugagccugggagcu	Enzyme activity	TriLink
18 mer reverse complement (fluorescence)	AGCTCCCAGGCTCAGATC	Enzyme activity	Lofstrand Labs
1R hairpin-DNA duplex	GACACcTGATTCGG/iBio-dT/AGAATCAGGTGT	SPR analysis	IDT
